# Value assessment of NMPA-approved new cancer drugs for solid cancer in China, 2016–2020

**DOI:** 10.3389/fpubh.2023.1109668

**Published:** 2023-02-24

**Authors:** Jing Luo, Shunlong Ou, Hua Wei, Xiaoli Qin, Rui Peng, Song Wang, Qian Jiang

**Affiliations:** ^1^School of Medicine, University of Electronic Science and Technology of China, Chengdu, Sichuan, China; ^2^Department of Pharmacy, Sichuan Clinical Research Center for Cancer, Sichuan Cancer Hospital & Institute, Sichuan Cancer Center, Affiliated Cancer Hospital of University of Electronic Science and Technology of China, Chengdu, Sichuan, China; ^3^Department of Pharmacy, Chengdu Second People's Hospital, Chengdu, Sichuan, China; ^4^Department of Pharmacy, The Third People's Hospital of Chengdu, Chengdu, Sichuan, China

**Keywords:** cancer drugs, clinical benefit, cost, ASCO-VF, ESMO-MCBS

## Abstract

**Background:**

Whether the high cost of cancer drugs is commensurate with their value to patients, which has become the focus of public concern. We aimed to assess the value of new cancer drugs approved for solid cancer in China and to explore the association between price and value of drugs.

**Methods:**

We identified all new drugs for solid tumor that approved by the China's National Medical Products Administration (NMPA) between 2016 and 2020. The value of these drugs was assessed according to the American Society of Clinical Oncology Value Framework (ASCO-VF) and the European Society for Medical Oncology Magnitude of Clinical Benefit Scale (ESMO-MCBS). We calculated *Cohen's* κ statistic to describe agreement between the two frameworks. Spearman's correlation coefficient was used to evaluate the correlation between price and value of drugs.

**Results:**

Between 2016 and 2020, 37 new drugs were approved by the NMPA for solid tumor and we could evaluate the value of 28 drugs (76%). Eight (29%) of drugs were approved for non-small-cell lung cancer and 6 (21%) for breast cancer. ASCO-VF scores had a range of −20 to 110.1, and the median score was 43.3 (inter-quartile range 27.1–58.35). Only seven drugs (25%) met the ASCO-VF cutoff score. By the ESMO-MCBS, 13 drugs showed a meaningful value. Agreement between these two frameworks thresholds was only fair (κ = 0.515, *P* < 0.05). We found no statistically significant correlation between launch price of drugs and clinical benefit according to both frameworks.

**Conclusions:**

Not all NMPA-approved new cancer drugs had meaningful value as measured by ASCO-VF or ESMO-MCBS. There was no significant correlation between drug price and the level of clinical benefit.

## Introduction

Innovations in cancer therapy, particularly the influx of new drugs have yielded high expectations of transform treatment of the disease from all healthcare stakeholders (1, 2). Nevertheless, dramatic rise in drug costs has recently highlighted a vigorous debate over whether cancer drugs prices, especially for that of targeted drugs and immunotherapies, commensurate with their value to patients, within reach of who need them not only in developed country, but also in developing country like China with scarce resources and rising demand for health services (3, 4).

To our knowledge, not all people know the price of everything but the value of nothing. It has never been more important to assess the value of new cancer drugs, and several organizations including the American Society of Clinical Oncology (ASCO), European Society for Medical Oncology (ESMO), Institute for Clinical and Economic Review (ICER), National Comprehensive Cancer Network (NCCN), and the pan-Canadian Oncology Drug Review Expert Review Committee (pCODRERC) have recently taken a step forward in this endeavor, developing tools for value assessment (5–11). All these tools have been designed for value assessment with the aim of weighing up the balance between efficacy, toxicity, quality of life and costs. Despite of their different conceptual definition of “value,” Bentley et al. (12) reported that the ASCO and ESMO tools demonstrated convergent validity and inter-rater reliability for value assessment for new cancer drugs.

In recent years, regulatory reforms have led to the introduction of a series of expedited programs to accelerate development, review, and approval of new drugs in China (13). Here, we overview the landscape of new cancer drugs approved by NMPA for solid cancer between 2016 and 2020 in China, describe the value of these drugs and further explore whether value is related to the drug price.

## Methods

### Data sources and extraction

We used the publicly available data to identify all new drugs (new molecular entities and novel biologic agents) approved by the China's National Medical Products Administration (NMPA) between January 1, 2016 and December 31, 2020, with initial indications for solid tumor. Meanwhile, we assessed whether the drug was granted with one of expedited programs in NMPA pathways and designations to accelerate drug approval (special review, priority review, conditional approval, urgently needed overseas drugs, and breakthrough therapy). Notably, drugs that were later approved for additional indications were not considered in this study.

The launch price and postlaunch price of drugs were extracted from the trade name and generic name recorded in the Hospital Information System (HIS). To estimate monthly treatment cost of a drug, we used the prescription and dosing information from the NMPA-approved label. Monthly treatment costs were calculated over an average of 30 days on the basis of the dosage schedule for an adult patient weighing 60 kg with a body surface area of 1.70 m^2^. The cost of all regimes was adjusted to provide the price per 4-week period (33.3% increase for 3-week treatment cycles and 100% increase for 2-week treatment cycles). Drug prices were converted to US dollars at the exchange rate as of August 29, 2022.

To quantify the clinical benefit from the pivotal clinical trials supporting regulatory approval, we applied two value frameworks developed by ASCO and ESMO, namely the American Society of Clinical Oncology Value Framework (ASCO-VF) version 2 (6), and European Society for Medical Oncology Magnitude of Clinical Benefit Scale (ESMO-MCBS) version 1.1 (8). Scores were assessed by one reviewer and checked by a second one, with any discrepancies resolved by a senior reviewer. In contrast to ESMO-MCBS, ASCO-VF was not planned to score single-arm studies and was therefore only suitable for phase II or III randomized clinical trials. In cases in which multiple pivotal clinical trials have been done and yield different clinical benefit scores for a given drug, the highest score was considered. Consistent with the developer of the value frameworks, meaningful clinical benefit was defined as a grade of A or B (for the curative setting) or 4, 5 (for the palliative setting) using ESMO-MCBS, whereas ASCO-VF did not clearly define what score was deemed “meaningful value.” Cherny et al. (14) recommended that the optimal threshold score of 45 or higher was proposed for recognizing substantial benefit for ASCO-VF by generating receiver operating characteristic (ROC) curves. Nevertheless, given the differences in construction and goals of ASCO-VF and ESMO-MCBS, they might yield some discordance in a cohort of studies. Thus, we split scores at the 75th percentile of ASCO-VF scores as the cutoff score for subsequent analyses, referring to the meaningful value achieved of ESMO-MCBS as a grade of 4, 5, B, or A (15).

### Statistical analysis

All data were collected in an Excel file designed for this study. Statistical analysis was conducted in IBM SPSS 25.0. Continuous data were graphed and analyzed to assess the normality of the underlying distribution. Spearman's correlation coefficient was used to describe the association between launch prices and clinical benefit according to ESMO-MCBS and ASCO-VF. We generated a ROC curve to establish a discrimination threshold of ASCO-VF scores to meet ESMO-MCBS criteria. *P* < 0.05 was deemed statistically significant.

## Results

### Number and characteristics of new drugs

**Figure 1:**
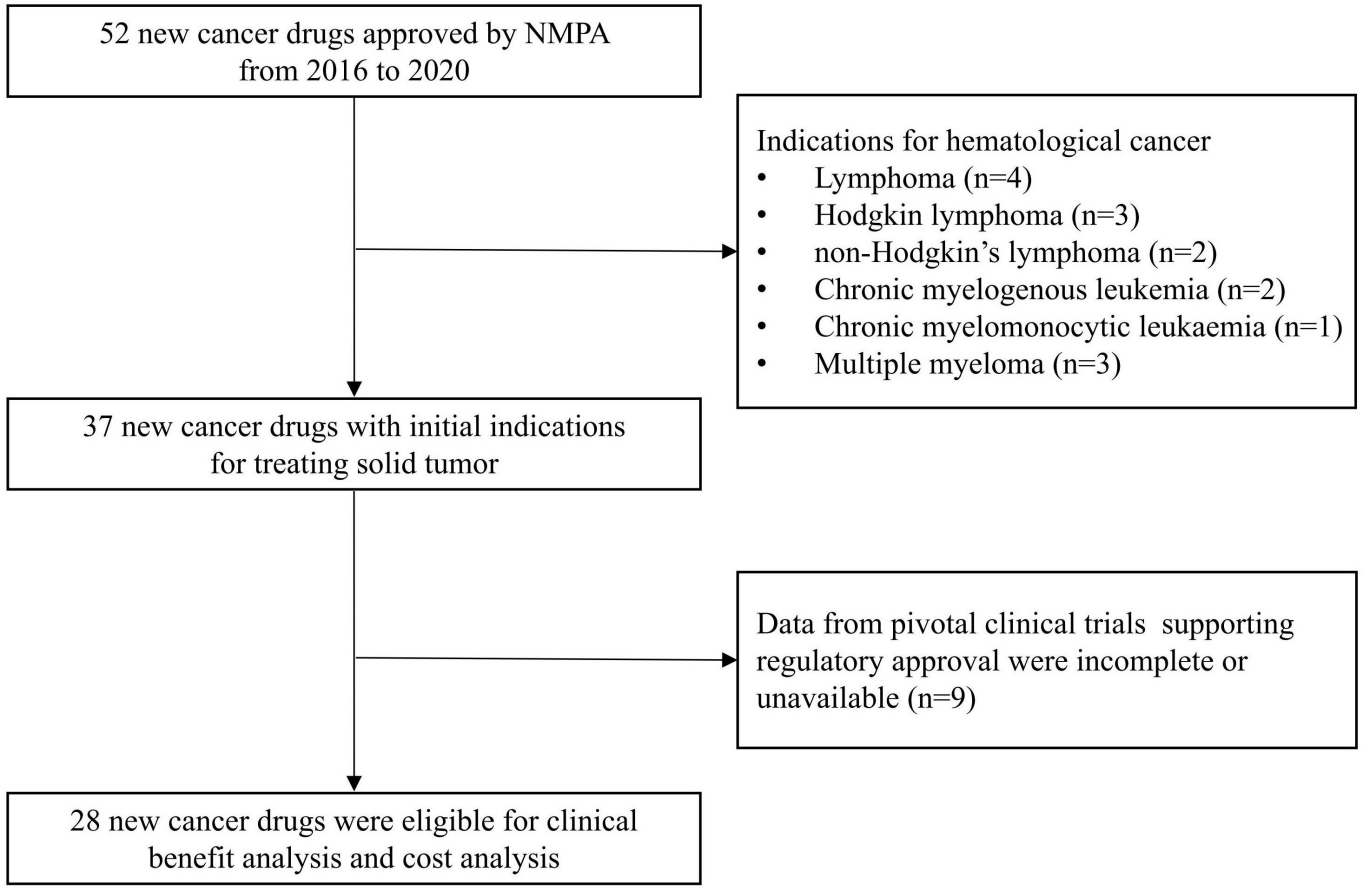
Identification of sample new cancer drugs approved by NMPA for treating solid tumor.

From 2016 to 2020, 52 new cancer drugs received initial regulatory approval by NMPA, 37 (71%) of which were approved for treating solid tumors and 15 (29%) were approved for treatment of hematologic cancers (Figure 1). Because data from pivotal clinical trials of nine drugs were incomplete or unavailable, only 28 drugs with prices and pivotal trials data were analyzed for subsequent analyses. The most common indications were non-small-cell lung cancer (*N* = 8, 29%) and breast cancer (*N* = 6, 21%) (Table 1). Of these, 23 drugs were imported from abroad and five drugs were domestic. Furthermore, 24 of which have benefited from at least one expedited program and most received priority review and special review.

**Table 1:** Characteristics and clinical benefit of NMPA-approved new drugs for treating solid tumor from 2016 to 2020 using the ASCO-VF and the ESMO-MCBS.

**Brand name**	**Generic name**	**Year**	**Initial indication approved by NMPA**	**Origin**	**Indication listed in NRDL**	**Expedited programs**	**ESMO-MCBS grade**	**ASCO-VF score**
Tagrisso	Osimertinib	2017	Non-small-cell lung cancer	Imported	Yes	PR, SR	5	110.1
Stivarga	Regorafenib	2017	colorectal cancer	Imported	Yes	PR, SR	3	25
Zelboraf	Vemurafenib	2017	Melanoma	Imported	Yes	PR	2	63
Gilotrif	Afatinib	2017	Non-small-cell lung cancer	Imported	Yes	PR, SR	2	−2.4
Votrient	Pazopanib	2017	renal-cell cancer	Imported	Yes	None	3	28.6
Keytruda	Pembrolizumab	2018	Melanoma	Imported	No	UNOD	4	45.9
Alecensa	Alectinib	2018	Non-small-cell lung cancer	Imported	Yes	PR, SR	4	47.6
Focusv	Anotinib	2018	Non-small-cell lung cancer	Domestic	Yes	PR, SR	NA	−20
Ibrance	Palbociclib	2018	Breast cancer	Imported	No	PR, SR	3	23.2
Irene	Pyrotinib	2018	Breast cancer	Domestic	Yes	PR, CA, SR	3	54.7
Elunate	fruquintinib	2018	colorectal cancer	Domestic	Yes	PR	3	8
Lenvima	Lenvatinib	2018	hepatocellular cancer	Imported	Yes	PR, SR	2	25.6
Perjeta	Pertuzumab*	2018	Breast cancer	Imported	Yes	None	B	36.6
Perjeta	Pertuzumab*	2018	Breast cancer	Imported	Yes	None	4	32
Zykadia	Ceritinib	2018	Non-small-cell lung cancer	Imported	Yes	PR, SR	1	30.8
Lynparza	Olaparib	2018	Ovarian cancer	Imported	Yes	PR, SR	3	62
Opdivo	Nivolumab	2018	Non-small-cell lung cancer	Imported	No	UNOD	4	43.3
Imfinzi	Durvalumab	2019	Non-small-cell lung cancer	Imported	No	None	3	71.5
Vizimpro	Dacomitinib	2019	Non-small-cell lung cancer	Imported	Yes	PR, SR	4	39.6
Xtandi	Enzalutamide	2019	Prostate cancer	Imported	Yes	PR, SR	4	43
Tafinlar	Dabrafenib	2019	Melanoma	Imported	Yes	PR, SR	A	34.4
Zejula	Niraparib	2019	Ovarian cancer	Domestic	Yes	PR, SR	3	64.4
Lonsurf	Trifluridine and Tipiracil Hydrochloride	2019	Colorectal cancer	Imported	No	SR	2	25.5
Erleada	Apalutamide	2019	Prostate cancer	Imported	Yes	UNOD	3	43.6
Cipterbin	Inetetamab	2020	Breast cancer	Domestic	Yes	PR	5	81.2
Kadcyla	Trastuzumab Emtansine	2020	Breast cancer	Imported	No	PR	A	68
Xofigo	Radium-223	2020	Prostate cancer	Imported	No	PR	5	44.3
Nerlynx	Neratinib	2020	Breast cancer	Imported	Yes	SR	A	53.7
Tecentriq	Atezolizumab	2020	Small cell lung cancer	Imported	No	None	2	49.8

NMPA, National Medical Products Administration; ASCO-VF, American Society of Clinical Oncology Value Framework; EMSO-MCBS, European Society for Medical Oncology Magnitude of Clinical Benefit Scale; NRDL, National Reimbursement Drug List; PR, priority review; SR, special review; UNOD, urgently needed overseas drugs; NA, not applicable.

^*^Pertuzumab had two therapies (the curative setting and the palliative setting).

### Clinical benefit of new drugs

For new drugs used for treating solid tumors, the median ASCO-VF score was 43.3 (interquartile range, 27.1–58.35; range −20 to 110.1), and the scores were normally distributed (Supplementary Figure S1). 14 drugs fell below, 14 drugs were above. We split scores at the 75th percentile of ASCO-VF scores-−58.35 as the cutoff score that deemed “meaningful clinical benefit”. Seven drugs were above the threshold whereas 21 (75%) fell below. By the ESMO-MCBS, 13 drugs met the criteria for meaningful benefit. Three (27%) of the 13 drugs meeting ESMO-MCBS thresholds were above the 75th percentile of ASCO-VF scores −58.35. For drugs in the palliative setting, Of the 19 drugs that did not meet the ASCO-VF cutoff score, only 12 fell below the ESMO-MCBS criteria. For drugs in curative setting, four (100%) of four drugs met ESMO-MCBS thresholds, only one were above the ASCO-VF cutoff score. The clinical benefit was shown in Table 1.

### Association between ASCO-VF and ESMO-MCBS

ROC curve was used to establish a discrimination threshold for ASCO-VF score to meet the ESMO-MCBS criteria of meaningful clinical benefit, and the threshold was determined to be approximately 31. However, the area under the curve was 0.662, suggesting only fair predictive value (Supplementary Figure S2). Agreement between ASCO-VF and ESMO-MCBS thresholds was only fair (κ = 0.515, *P* < 0.05).

### Correlation between price and value of drugs

**Figure 2:**
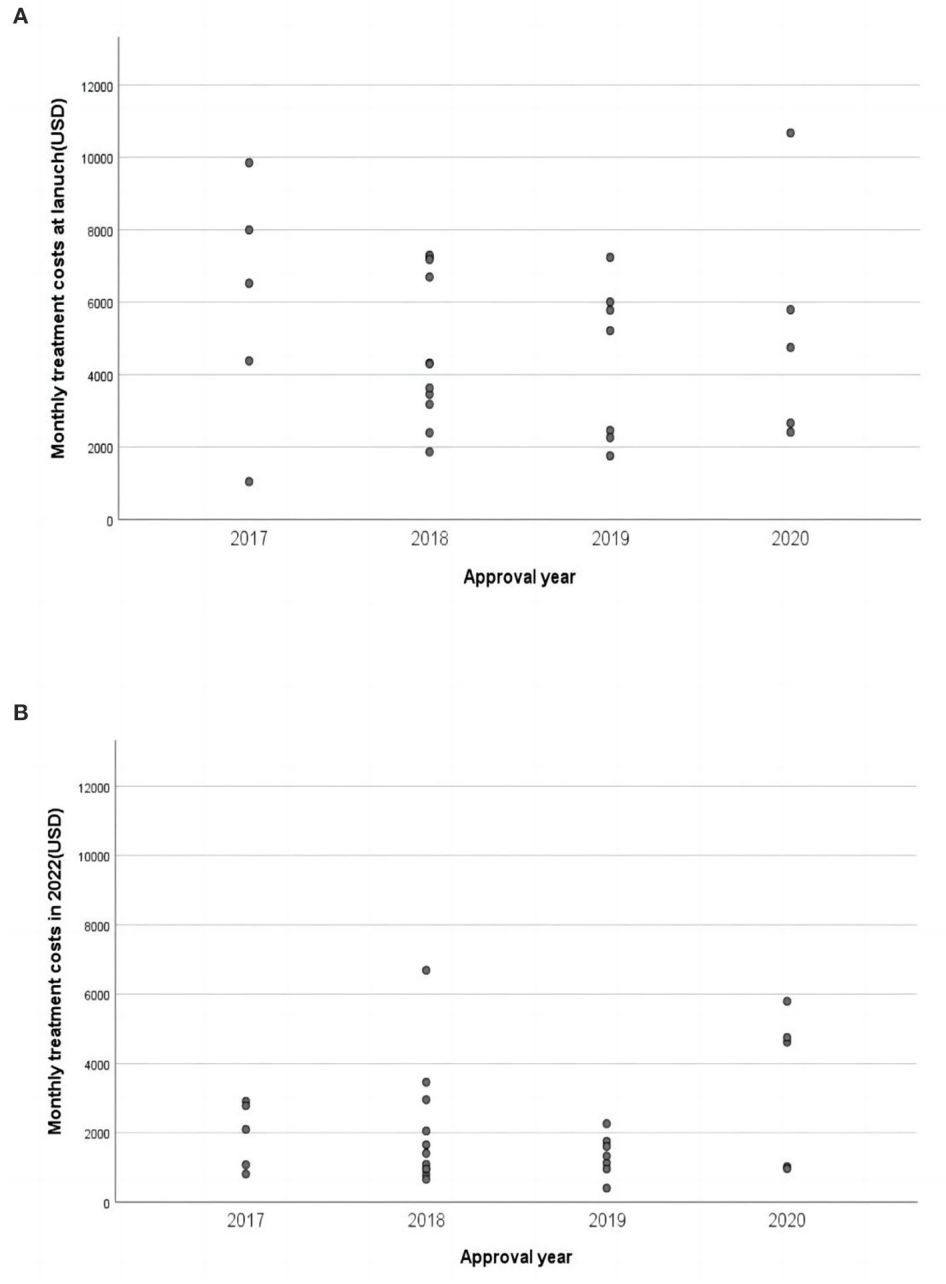
Monthly treatment costs of approved cancer drugs in China. **(A)** Monthly treatment costs of approved cancer drugs at launch. **(B)** Monthly treatment costs of approved cancer drugs in 2022.

**Figure 3:**
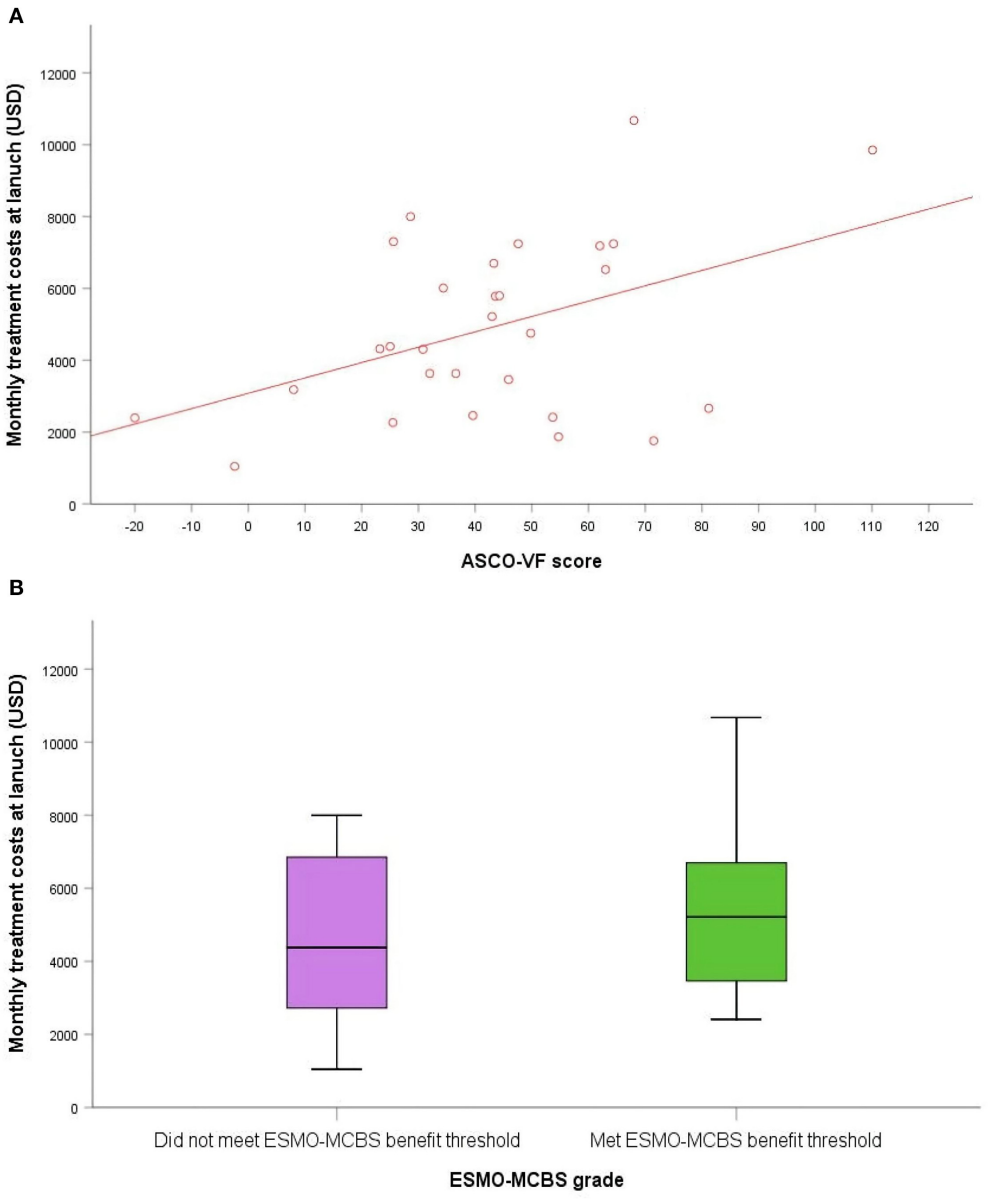
The association between clinical benefit and costs of approved cancer drugs in China. **(A)** Scatterplot of association between ASCO-VF scores and monthly treatment costs. **(B)** Box plot of association between ESMO-MCBS grades and monthly treatment costs.

In China, the median monthly treatment costs per patient at launch for the included cancer drugs were $4,381. As of August 25, 2022, the median monthly treatment costs were $1,408, indicating that the postlaunch price changes for most NMPA approved cancer drugs were roughly three times less than the launch prices (Figure 2). We found no statistically significant associations between launch prices of drugs approved for solid tumors and clinical benefit were observed according to both frameworks (Figure 3). For ASCO-VF, launch prices had weak correlation with clinical benefit (Spearman's ρ < 0.30; *P* > 0.05). The launch price of new cancer drugs and ESMO-MCBS grades had weak correlation (Spearman's ρ < 0.30; *P* > 0.05).

## Discussion

To the best of our knowledge, this is the first study in China to comprehensively evaluate the value of new cancer drugs using ASCO-VF and ESMO-MCBS, and to investigate the correlation between price of new drugs and their clinical benefits. In our review of all new cancer drugs approved by NMPA for solid cancer between 2016 and 2020, approximately half of new drugs achieved meaningful clinical benefit according to ESMO-MCBS. We found that all new drugs had a wide range of ASCO-VF scores and only fair association between ASCO-VF and ESMO-MCBS, which was consistent with previous studies (15, 16).

About three-quarters of new drugs were listed in the National Reimbursement Drug List (NRDL). In contrast to the increasing prices of cancer drugs in the years after approval in the US, the daily treatment cost of cancer agents has fallen in China, especially for targeted therapies and branded products (17). However, we found no significant correlation between price and clinical benefit according to the two frameworks. With rising demand for health services in China, prices should be better aligned with value, especially for expensive cancer drugs. Nearly half of cancer drug indications approved in China had shown OS benefit (18). Lack of a clear association between price and clinical benefit indicates that value frameworks can help not only identify drugs with low or uncertain clinical benefit that should be targeted for price negotiations, but also therapies with evidence of higher clinical benefit to improve access to benefit drugs (19, 20).

In 2015, China's government proposed to establish an open and transparent price negotiation mechanism with multi-party participation for some patented, high-priced drugs and exclusively produced drugs. In the same year, the first round of national-level drug pricing negotiations was launched (21). The dimensions of national price-negotiation cancer drugs include the value of drugs and the affordability of China's healthcare system funds. China's government has sufficient bargaining power. Since 2017, the government has started annual centralized price negotiation, which has sharply fallen drug prices compared with launch prices, resulting in increased affordability of expensive cancer drugs (22). However, as new clinical trials were conducted, results of post-approval clinical trials might lead to dynamic changes in value frameworks scores. Moreover, the qualifications of medical insurance payment tend to be strict. For new indications of cancer drugs, the out-of-pocket spending of patients have not been reduced, leading to increase the financial toxicity of patients. Therefore, the government needs to routinely monitor the impacts of shifts in medicine on resource utilization.

This study has several limitations. Most breakthrough-designated drugs were approved based on single-arm or non-randomized trials, resulting in the level of evidence for breakthrough therapies was inferior to that for non-breakthrough therapies when assessing the value through value frameworks (23). Thence, it is highly unfair to assess clinical benefit in this situation. Furthermore, treatment effects are known to be heterogeneous, and some patients can benefit greatly from drugs with a low value score (24). This was one of the limitations of the study, which did not evaluate the value of all new drugs. It is complicated to precisely define value of a drug, and our assessment relied entirely on data reported of clinical trial, not taking into account other factors that may influence value of a drug. Secondly, the association between ASCO-VF and ESMO-MCBS was only fair based on our findings. The ASCO-VF and the ESMO-MCBS have shared the goal of assisting clinicians and patients to measure the relative benefits of new cancer drugs (23). Nevertheless, due to the differences in the frameworks' inherent designs, especially the method and indicators of the frameworks differ greatly, resulting in greatly divergences in scores and grades (14, 16). In addition, we used scores at the 75th percentile of ASCO-VF scores as a threshold for comparisons. Nevertheless, changing this cutoff score will influence the degree of correlation between the two value frameworks. Meanwhile, we did not consider the duration of treatment when calculating the costs of a drug. But most of drugs in the palliative setting, so as long as response to treatment continues, monthly drug costs could be used. Furthermore, we did not investigate whether all cancer drugs that were approved by the NMPA were also approved in other countries. Therefore, our study needs to be followed by further study to assess the value in other countries especially emerging or developing countries comprehensively, promoting the situation of other countries in terms of access to oncology medicines with value assessment.

## Conclusion

In summary, ASCO-VF and ESMO-MCBS are important tools for assessing value of cancer drugs, although the correlation between these frameworks is fair. Based on the available evidence, not all new drugs met the meaningful threshold according to ASCO-VF or ESMO-MCBS. The price of a drug was not significantly related to the level of clinical benefit, and the cost could not justify its value. Policy makers requires to improve the alignment between drug prices and clinical benefits in order to provide optimal cancer treatments for patients.

## Data availability statement

The original contributions presented in the study are included in the article/Supplementary material, further inquiries can be directed to the corresponding author.

## Author contributions

Conceptualization and writing—review and editing: JL and QJ. Methodology: JL and SO. Software and writing—original draft preparation: JL. Data curation: HW, XQ, and RP. Formal analysis: JL, SO, and SW. Visualization: HW and XQ. Supervision and funding acquisition: QJ. All authors have read and agreed to the published version of the manuscript.
