# Supplementary material for: Value assessment of NMPA-approved new cancer drugs for solid cancer in China, 2016–2020
Source: Front Public Health. 2023 Feb 24;11:1109668. doi: 10.3389/fpubh.2023.1109668 (PMC9998930; doi:10.3389/fpubh.2023.1109668)

**Supplementary Content**

Figure S1 Histograms for the distributions of the ASCO-VF Scores.

Figure S2 Receiver Operating Characteristic curve for ASCO-VF and ESMO-MCBS among new drugs for treating solid tumor that approved by NMPA between 2016 and 2020.

Figure S1 Histograms for the distributions of the ASCO-VF Scores.


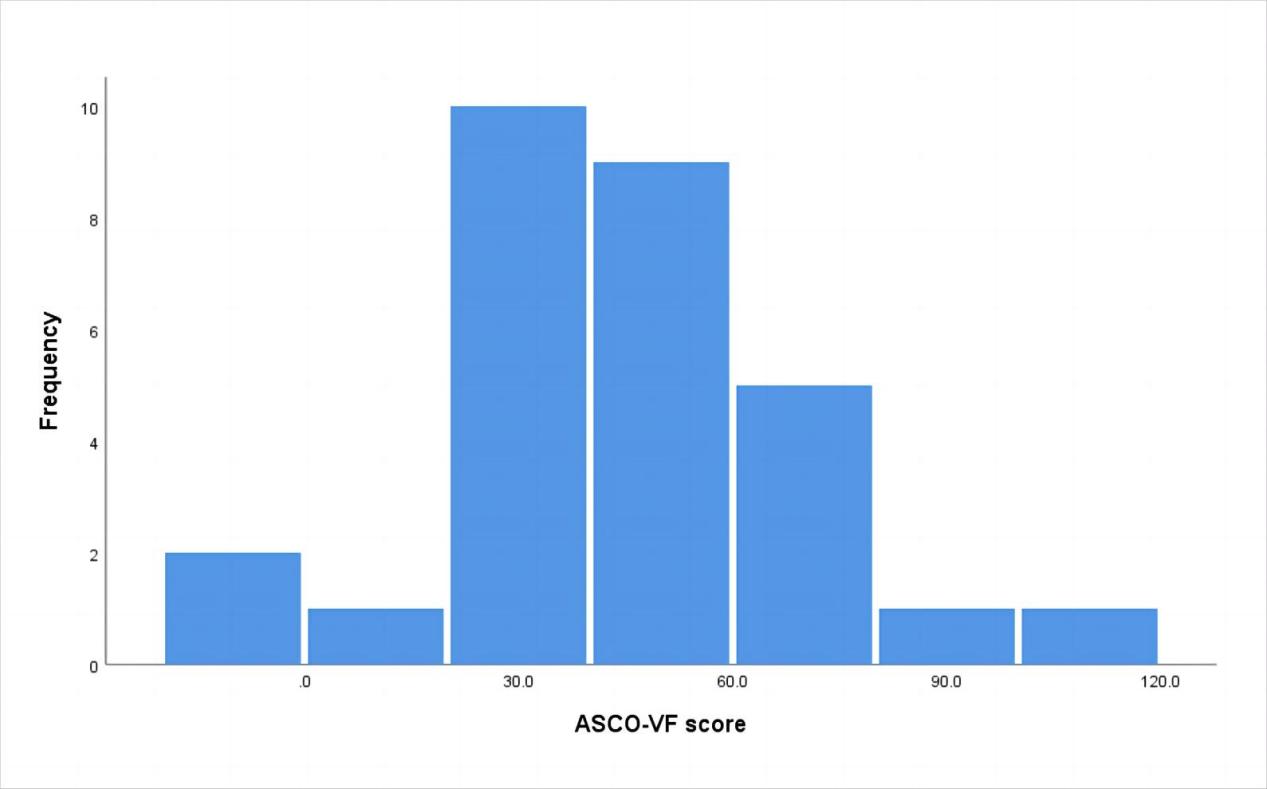


Figure S2 Receiver Operating Characteristic curve for ASCO-VF and ESMO-MCBS among new drugs for treating solid tumor that approved by NMPA between 2016 and 2020.


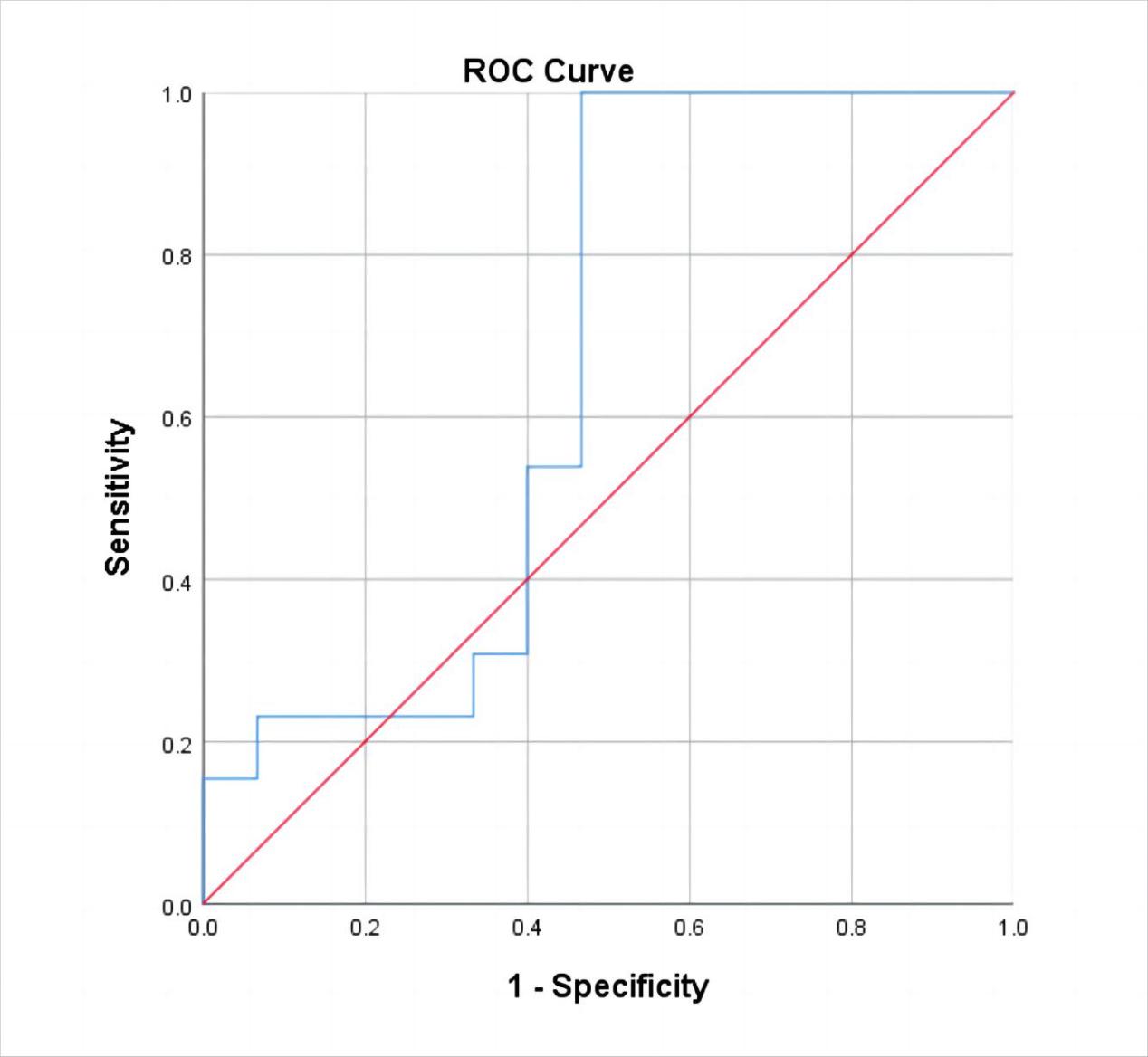

Supplement: Supplementary file 1 [file Table_1.DOCX]
